# The exploration of the potential mechanism of oxymatrine-mediated antipruritic effect based on network pharmacology and weighted gene co-expression network analysis

**DOI:** 10.3389/fphar.2022.946602

**Published:** 2022-09-23

**Authors:** Zhenhui Luo, Tingting Zhao, Mengqin Yi, Tingting Wang, Zhenglang Zhang, Wenbin Li, Na Lin, Shangdong Liang, Alexei Verkhratsky, Hong Nie

**Affiliations:** ^1^ Guangdong Province Key Laboratory of Pharmacodynamic Constituents of TCM and New Drugs Research, College of Pharmacy, Jinan University, Guangzhou, China; ^2^ International Cooperative Laboratory of Traditional Chinese Medicine Modernization and Innovative Drug Development of Chinese Ministry of Education (MOE), College of Pharmacy, Jinan University, Guangzhou, China; ^3^ Institute of Chinese Materia Medica, China Academy of Chinese Medical Sciences, Beijing, China; ^4^ Neuropharmacology Laboratory of Physiology Department, Basic Medical School, Nanchang University, Nanchang, China; ^5^ Faculty of Biology, Medicine and Health, the University of Manchester, Manchester, United Kingdom

**Keywords:** oxymatrine, itching, inflammation, WGCNA, network pharmacology

## Abstract

The treatment of chronic itch is considered to be a challenge for its non-histamine dependence and the search for alternative medicine is still striving. The pathology of the chronic itch is closely related to immune system regulation and inflammatory response. Oxymatrine (OMT) is a traditional Chinese medicine ingredient extracted from the roots of *Sophora flavescens* Aiton with significant antitumor, analgesic, and anti-inflammatory effects. However, the underlying mechanism of OMT on chronic itch is obscure, which limits clinical application. Hence, this study is aimed to clarify the pruritus alleviation mechanism of OMT by combining network pharmacology analysis, weighted gene co-expression analysis (WGCNA), and molecular docking. We screened 125 common targets of OMT regulating inflammation and pruritus with pharmacology technology, the GO enrichment function analysis and KEGG signaling pathway analysis to demonstrate the close relation to the signaling pathways regulating inflammation such as MAPK signaling pathway and PI3K-AKT signaling pathway. We adopted the most relevant templates for pruritus diseases, combined with network pharmacology to preliminarily screen out 3 OMT functions and regulatory targets, exerting a good connection and correlation with the target at the screened disease targets. Further experiments were conducted to explore the potential mechanism of OMT using the LPS-induced RAW264.7 cell inflammation model. The results showed that pretreatment with different concentrations of OMT (25 μM, 50 μM, and 100 μM) for 24 h, inhibited expression of IL-6, iNOS TLR4 and TGFR-1 as well as apoptosis of Raw264.7 cells induced by LPS. Moreover, OMT effectively inhibited LPS-induced MAPK pathway activation and the expression of related sites MAP2K1, MAPK8 MAP2K4, and MAPKAP-K2 in RAW 264.7 cells. The OMT also reduced the phosphorylation of p-38, associated with site in the activation of MAPK signaling pathway. These results could contribute to a better understanding of the mechanisms underlying how OMT alleviates inflammation to treat chronic pruritic diseases and provide a potential drug for the treatment of chronic itch.

## Introduction

Itching is a typical side effect of skin conditions, which was initially defined as a feeling that may inspire the desire to scratch by Samuel Hafenreffer in 1660 (Bernhard, 2005). Chronic itching has no effective treatment, which significantly influences patients’ quality of life and physical and mental health (Pereira and Stander, 2019). About 280 million individuals worldwide (4% of global population) suffer from pruritus, with those suffering from severe pruritus accounting for 1% of the overall population (Matterne et al., 2013; Nutten, 2015). Many chronic diseases, including most skin diseases (allergic contact dermatitis, psoriasis, etc.), neurological diseases (multiple sclerosis, herpes zoster, etc.), cancers, and mental problems, are associated with long-term skin itching (Kiebert et al., 2002; Rishe et al., 2008; Reich et al., 2016).

Antihistamines, hormone medications, anticonvulsants, antidepressants, immunosuppressants, and other systemic therapy agents are commonly used to treat intractable pruritus in more severe cases. However, the majority of these medications have substantial side effects, such as sleepiness, gastrointestinal reactions, raised or lowered blood pressure, upper respiratory tract infection, mental problems, and so on (Stander et al., 2007). Patients taking these medications for a long period have been suffering from these side effects for years. As a result, society must understand the cause of intractable pruritus and create specific antipruritic medications that are safe, effective, and non-toxic.

*Sophora flavescens* (SF) is a typical traditional Chinese medicine (TCM) widely used in China to relieve itch through oral administration in tablet, capsule and decoction, or topical application (He et al., 2015). Previous pharmacological studies found that SF contains various components, mainly alkaloids, flavonoids, and polysaccharides, exerting various biological activities including antibacterial, anti-inflammatory, and immunomodulatory (Li et al., 2020; You et al., 2020). In addition, some vital compounds of SF, such as oxymatrine (OMT), have been reported to have antipruritic activity (Xiang et al., 2020). However, the potential mechanism of action by which bioactive compounds in SF can treat histamine-independent pruritus has not been fully elucidated.

OMT, a weakly basic quinolizidine alkaloid produced from the roots of *Sophora flavescens* Aiton [Fabaceae], is a white or off-white crystalline powder with a bitter taste that is soluble in water, methanol, ethanol, chloroform, and benzene. It has the chemical formula C_15_H_24_N_2_O_2_, with a molecular weight of 264.36, and a molecular structure that is depicted in Figure 2. OMT possesses a range of biological effects, including antipruritic (Xu et al., 2018; Zhu et al., 2020), antiviral (Jiang et al., 2017), anticancer (Halim et al., 2019), and antifibrosis (Liu et al., 2020). In addition, OMT can improve ischemia-reperfusion injury and has cardioprotective, hypoglycemic, hypolipidemic and sedative, analgesic, anti-epileptic, and other central nervous system effects (Liu et al., 2012; Guo et al., 2014; Zhou et al., 2017; Wang et al., 2022). Moreover, OMT exhibits anti-pruritic and anti-inflammatory effects in ACD mice by regulating inflammatory mediators and restoring Th1/Th2 and Th17/Treg immunological balance, according to prior research (Xu et al., 2018). However, its effect on innate immune cells and on neutrophils and macrophages remain unknown, necessitating more investigation.

**FIGURE 1:**
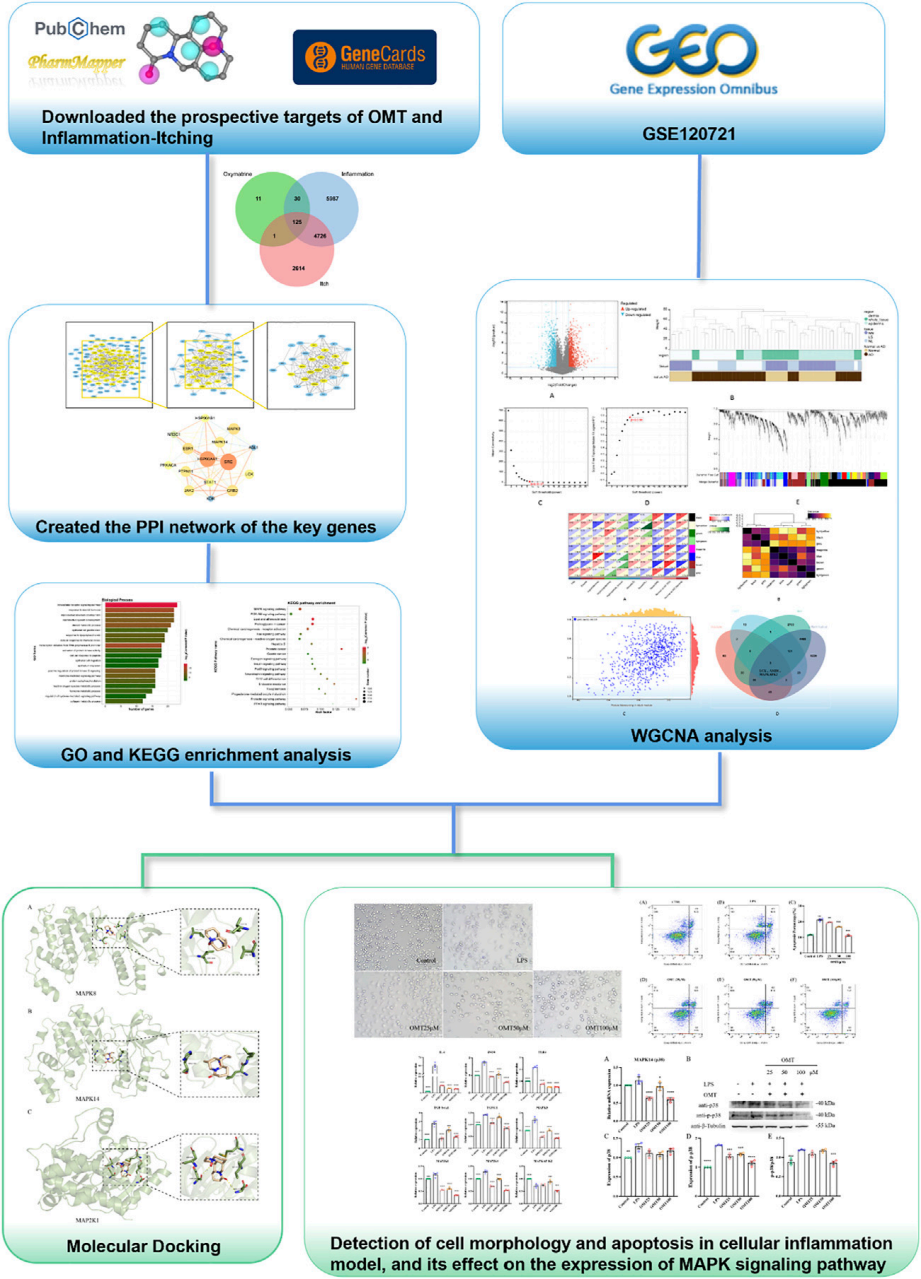
Thu flow chart of this slimly aims to investigate the potential mechanism of action of OMT in the liniment of pruritus.

Therefore, this study utilized a multi-dimensional network and experimental verification to reveal the biological mechanism of OMT’s antipruritic activity and to provide a scientific foundation for OMT clinical trial research and commercial development. The technical methods and experimental results for evaluating the effect and mechanism of OMT on chronic pruritus are shown in the flowchart in Figure 1.

## Materials and methods

### Network pharmacology analysis data preparation

The molecular structure file of OMT was downloaded from PubChem (https://pubchem.ncbi.nlm.nih.gov/) (Kim et al., 2019) and uploaded to the PharmMapper (http://www.lilab-ecust.cn/pharmmapper/) (Liu et al., 2010) database’s target prediction tool to screen for potential OMT interaction targets, and the gene name matching to the target protein was acquired using the Uniprot protein database (https://www.uniprot.org/) (UniProt, 2021). The reported inflammation and pruritus-related genes were searched using the keywords “inflammation,” “pruritus,” and “itch” in the GeneCards database (https://www.genecards.org/, ver.4.9.0) (Safran et al., 2010), and the Venn diagram was utilized to screen the common targets with the prospective targets of OMT.

### OMT-inflammation-itching target network construction and analysis

The reported inflammation and pruritus-related genes were searched using the keywords “inflammation,” “pruritus,” and “itch” in the GeneCards database, and the Venn diagram was utilized to screen the common targets with the prospective targets of OMT. The common targets were loaded into the String database, the species was confined to “*Homo sapiens*,” the PPI network map was created, and the minimum needed interaction score was set to a high confidence level of 0.07, with the discrete targets concealed. The PPI network diagram was then fine-tuned using the Cytoscape statistical tools (version 3.7.0) (Doncheva et al., 2019).

### Enrichment analysis

#### GO analysis

We utilized the GO annotations of genes in the R package org for gene set functional enrichment analysis. Hs.eg.DB (version 3.1.0) as the background to mapping the genes to the background set using the R package cluster profile (version 3.14.3) to perform enrichment analysis to obtain gene set enrichment results. *p* values of 0.05 and an FDR of 0.25 were deemed statistically significant when the minimum gene set was 5 and the largest gene set was 5,000.

#### KEGG analysis

We utilized the KEGG rest API (https://www.kegg.jp/kegg/rest/keggapi.html) to retrieve the most recent gene annotations from the KEGG Pathway as a backdrop for gene set functional enrichment analysis. To obtain the results of gene set enrichment in the background set, the enrichment analysis was performed using the R software package cluster profile (version 3.14.3). *p* values of 0.05 and an FDR of 0.25 were deemed statistically significant when the minimum gene set was 5 and the largest gene set was 5,000.

### Weighted gene co-expression network analysis of disease-related potential target genes

GSE120721 (GPL570) data were obtained from the GEO database. We deleted genes with a standard deviation of 0 in each sample, removed outlier genes and samples using the good samples genes method of the R software package WGCNA, built a scale-free co-expression network with WGCNA, and Pearson all paired genes were conducted. Correlation matrix and mean linkage method, then use the power function Amn = |Cmn|^β (Cmn = Pearson correlation) to construct a weighted adjacency matrix between Gene m and Gene n (Amn = the adjacency between Gene m and Gene n) (Zhang and Horvath, 2005; Langfelder and Horvath, 2008). β was a soft-thresholding parameter that emphasized high gene-gene correlations while penalizing weak correlations. The adjacency was turned into a topological overlap matrix (TOM), which could quantify a Gene’s network connection defined as the sum of its adjacency with all other Genes for network generation, and the corresponding dissimilarity (1-TOM) was determined after choosing the power of 9. Average linkage hierarchical clustering was used to categorize Genes with comparable expression patterns into Gene modules using the TOM-based dissimilarity measure with a minimum size (Gene group) of 30 for the genes dendrogram and sensitivity set to 3. We estimated the dissimilarity of module eigengenes, set a cut line for the module dendrogram, and merged certain modules to further investigate the modules.

### Molecular docking simulation

OMT was obtained and downloaded from PubChem and converted OMT files to PDBQT format using Open Babel 2.4.1. Then we obtained and download the target protein’s crystal structure from the RCSB Protein Data Bank database (RCSB PDB, https://www.rcsb.org/) (Goodsell et al., 2020). The water and ligands were removed from the target protein with AutoDock Tools (Trott and Olson, 2010) and produced a new protein. It adds hydrogen atoms, calculates charge, exports the PDBQT format file, and determines the size and center of the docking box all at once. Vina was adopted to dock the active components with the target protein one by one and took the conformation with the highest docking score (Affinity). Finally, the results were analyzed with Pymol and created graphs.

### Cell culture and cell viability assay

RAW264.7 cells (Procell CL-0190, Wuhan Procell Life Technology Co., Ltd.) were grown in MEM-ALPHA culture medium (containing 10% FBS, 1% penicillin, and 1% streptomycin) at 37°C in a 5% CO_2_ incubator. The cytotoxicity of OMT on RAW264.7 cells was measured by CellCountingKit-8 (CCK-8, Dojindo). The cells were incubated for 24 h in 96-well plates. The cells were then co-treated for 6 h with different concentrations of OMT (0, 1, 2, 4, and 8 mM) (OMT, Aladdin, concentration ≥98%). 10 µL CCK-8 was added to each well and cultured. The well’s absorbance at 450 nm was monitored for another 1–4 h. The feasibility of the project was calculated. The following is how it was calculated: Cell viability = [(As-Ab)/(Ac-Ab)] × 100%. As: Absorbance of the experimental well (containing cells, culture medium, CCK8 solution, and drug solution), Ac: Absorbance of control wells (containing cells, culture medium, CCK8 solution), Ab: Absorbance of the blank well (containing medium, CCK8 solution).

### Quantitative real-time PCR

Total RNA was isolated from RAW264.7 cells using Trizol reagent, and the collected RNA was analyzed using a spectrophotometer for concentration and purity. RNA with an absorbance ratio of roughly 2.0 (OD260 nm/OD280 nm) was chosen for qPCR and transcribed into cDNA using RT Master Mix for qPCR II. Using a qPCR PreMix (SYBR Green) Kit, quantitative real-time PCR (qRT-PCR) was used to quantify the expression of the mRNA.Primers used in the test are shown in Table 1. The 2^−ΔΔCt^ method was used to calculate the relative expressions of the relevant genes.

**TABLE 1:** Primer sequences for RT-qPCR.

Name	Sequence (5'−3′)
iNOS(Sense sequence)	CAG​CTG​GGC​TGT​ACA​AAC​CTT
iNOS (Antisense sequence)	CAT​TGG​AAG​TGA​AGC​GTT​TCG
IL-6 (Sense sequence)	CTG​CAA​GAG​ACT​TCC​ATC​CAG
IL-6 (Antisense sequence)	AGT​GGT​ATA​GAC​AGG​TCT​GTT​CG
TLR4(Sense sequence)	GCA​TGG​CTT​ACA​CCA​CCT​CT
TLR4 (Antisense sequence)	GTC​TCC​ACA​GCC​ACC​AGA​TT
Tgfb1 (Sense sequence)	CTC​CCG​TGG​CTT​CTA​GTG​C
Tgfb1 (Antisense sequence)	GCC​TTA​GTT​TGG​ACA​GGA​TCT​G
Tgfbr1 (Sense sequence)	TCT​GCA​TTG​CAC​TTA​TGC​TGA
Tgfbr1 (Antisense sequence)	AAA​GGG​CGA​TCT​AGT​GAT​GGA
Mapk8(Sense sequence)	AGC​AGA​AGC​AAA​CGT​GAC​AAC
Mapk8 (Antisense sequence)	GCT​GCA​CAC​ACT​ATT​CCT​TGA​G
Mapkapk2(Sense sequence)	TTC​CCC​CAG​TTC​CAC​GTC​A
Mapkapk2 (Antisense sequence)	GCA​GCA​CCT​TCC​CGT​TGA​T
Map2k4(Sense sequence)	AAT​CGA​CAG​CAC​GGT​TTA​CTC
Map2k4 (Antisense sequence)	TGA​AAT​CCC​AGT​GTT​GTT​CAG​G
Map2k1(Sense sequence)	AAG​GTG​GGG​GAA​CTG​AAG​GAT
Map2k1 (Antisense sequence)	CGG​ATT​GCG​GGT​TTG​ATC​TC
Mapk14(Sense sequence)	#333333
	TGA​CCC​TTA​TGA​CCA​GTC​CTT​T
Mapk14 (Antisense sequence)	GTC​AGG​CTC​TTC​CAC​TCA​TCT​AT
GAPDH (Sense sequence)	GUA​UGA​CAA​CAG​CCU​CAA​GTT
GAPDH (Antisense sequence)	CUU​GAG​GCU​GUU​GUC​AUA​CTT

### Flow cytometry

The apoptosis of RAW264.7 cells induced by LPS induced by OMT was detected by flow cytometry. The cells in the six-well plate were pretreated with OMT and then LPS was added for co-culture for 24 h. After that, the cells in each group were harvested and subjected to two experiments. Wash with PBS, resuspended in 100 μL 1× Binding Buffer, add 2 μL 0.5 mg/ml 7-AAD and 2 μL L Annexin V-FITC solution, incubate for 15min at room temperature in the dark, add 300 μL^1^× Binding Buffer, mix well, transfer to 96-well plate, 1 h Fluorescence detection was performed by flow cytometry. (Annexin V-FITC has a maximum excitation light of 488 nm and an emission light of 520 nm. 7-AAD has a maximum excitation light of 488 nm and an emission light of 647 nm).

### Western blot

The procession of protein extraction in this study can be referred to the article (Luo et al., 2021). 20 µg per sample of proteins were separated by sodium dodecyl sulfate-polyacrylamide gel electrophoresis (SDS-PAGE) and deposited onto a polyvinylidene fluoride (PVDF) membrane after the concentration of proteins was determined. The membranes were blocked with 5% milk for 2 h before incubation with the primary antibody at 4°C overnight, which is as follow, Anti-p-38 (Affinity Biosciences, OH, United States), Anti-p-p-38 (Affinity Biosciences, OH, United States), β-Tubulin (Affinity Biosciences, OH, United States). After response with the primary antibody, the blotted PVDF membrane was treated with horseradish peroxidase (HRP) conjugated secondary antibod (Boster Biological Technology Co. Ltd.). The ECL chemiluminescence western blot detection technique was performed with a gel imaging system and the band intensity were analyzed *via* ImageJ.

### Statistical analysis

All data are presented as mean ± SEM of independent experiments. Statistical evaluation of the results was performed by one-way ANOVA and a *p-value* of 0.05 or less was considered statistically significant, GraphPad Prism 7 software was used for graphical analysis and visualization.

## Results

### Prediction of potential targets of OMT pharmacophore

**FIGURE 2:**
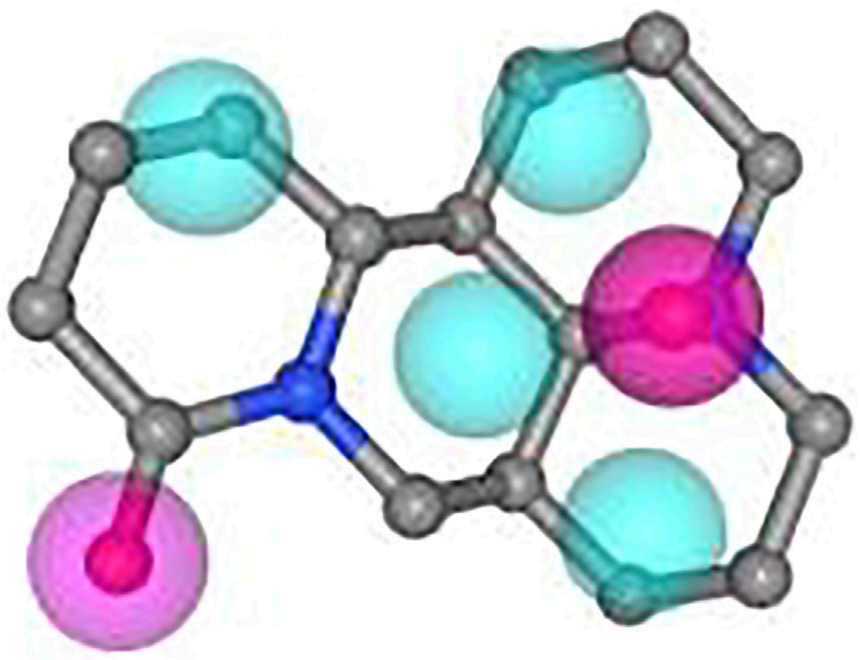
OMT pharznacopltre nail.

OMT has a total of six pharmacophores, the structure of which is depicted in Figure 2. The molecule was uploaded to the PharmMapper database for target prediction and was combined with the Uniprot database to obtain the corresponding gene name of the target protein. Table 2 presents the probable interaction targets with a score greater than 0.7, out of a total of 167 potential interaction targets found according to the pharmacophore.

**TABLE 2:** Potential mutual targets of OMT.

Serial number	Pharma model	Num feature	Fit	Norm fit	Uniprot	Gene symbol
1	1reu_v	3	2.894	0.9646	BMP2_HUMAN	BMP2
2	1w8l_v	3	2.804	0.9347	P62937	PPIA
3	1p49_v	3	2.794	0.9315	STS_HUMAN	STS
4	1e7a_v	3	2.772	0.9239	ALBU_HUMAN	ALB
5	2o65_v	3	2.765	0.9218	PIM1_HUMAN	PIM1
6	1j96_v	3	2.762	0.9207	AK1C2_HUMAN	AKR1C2
7	1rs0_v	3	2.685	0.8951	CFAB_HUMAN	CFB
8	3gam_v	3	2.662	0.8874	P16083	NQO2
9	1pmv_v	3	2.62	0.8734	MK10_HUMAN	MAPK10
10	1uki_v	3	2.582	0.8607	MK08_HUMAN	MAPK8
11	1l6l_v	3	2.4	0.7999	APOA2_HUMAN	APOA2
12	1if4_v	3	2.277	0.7591	CAH2_HUMAN	CA2
13	2ao6_v	4	2.96	0.74	ANDR_HUMAN	AR
14	1bm6_v	3	2.202	0.7341	MMP3_HUMAN	MMP3
15	1shj_v	3	2.201	0.7337	CASP7_HUMAN	CASP7
16	2ipw_v	4	2.927	0.7319	ALDR_HUMAN	AKR1B1
17	2fky_v	4	2.913	0.7283	KIF11_HUMAN	KIF11
18	2zas_v	4	2.912	0.7279	P62508	ESRRG
19	2of0_v	4	2.891	0.7228	BACE1_HUMAN	BACE1
20	1mx1_v	4	2.865	0.7162	EST1_HUMAN	CES1

### Collection of targets related to inflammatory and pruritic diseases

**FIGURE 3:**
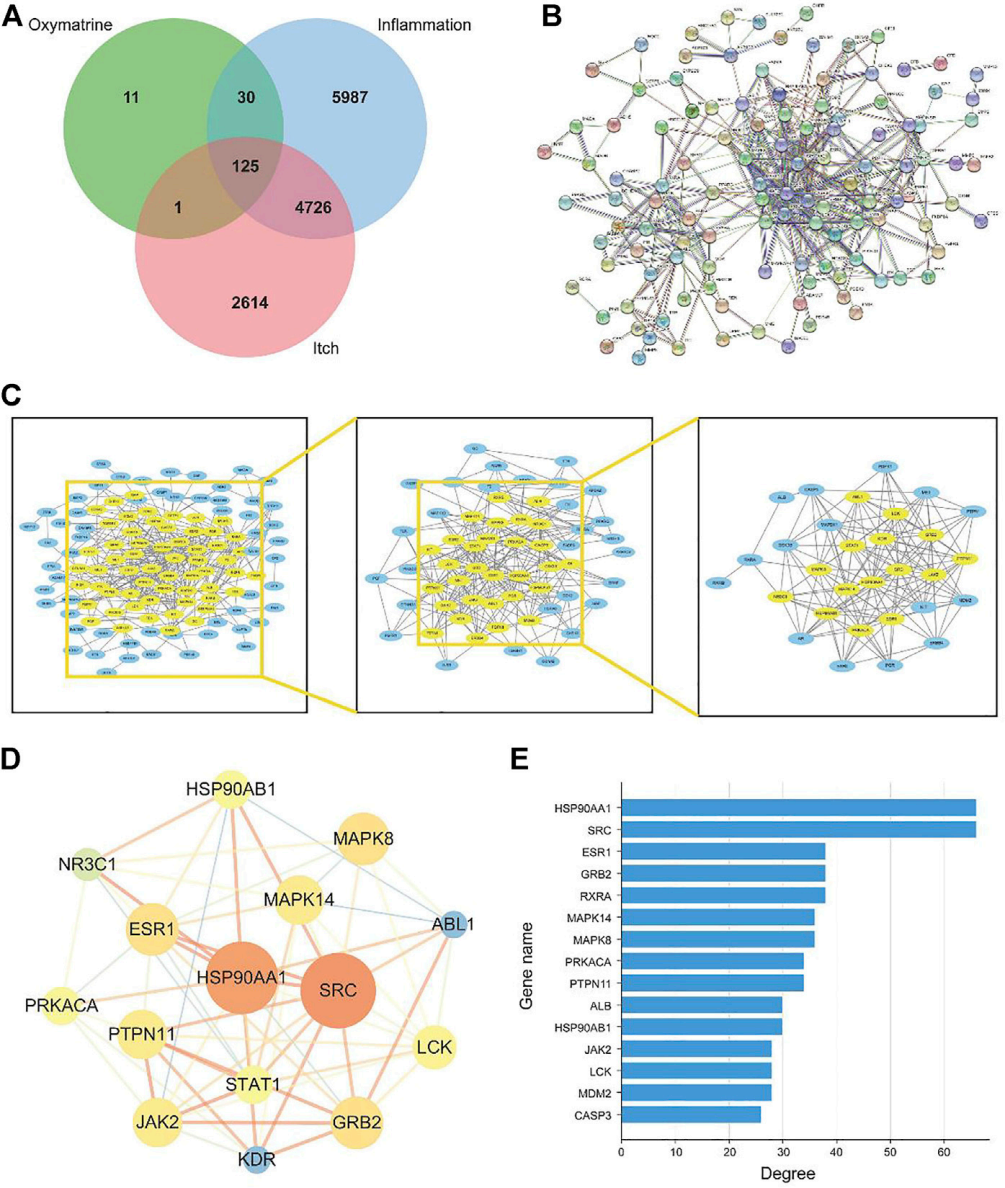
The construction and analysis of the intersection targets of OMT, inflammation, and itch. **(A)** Venn diagram m of OMT, itch, and inflammation **(B)** Protein-protein interaction (PPI) network of targets regulated by common targets of OMT, itch, and inflammation **(C)** Target network analysis diagram **(D)** Intersection target network diagram generated using Cytoscape. **(E)** Target degree value histogram of common targets of OMT, itch, and inflammation.

To understand the link between inflammation and itching, here we collected 10,868 “inflammation” related targets and found 7,466 reported pruritus-related genes inputting the keywords “pruritus” and “itch” and were used to screen OMT Co-acting targets of prospective targets through Venn diagrams as shown in Figure 3A. The estimated 156 OMT targets all lie within the targets of inflammation and pruritus, with 125 targets working together by the three, 30 of which were operating on OMT and inflammation alone. The target acting on OMT and pruritus alone is just one.

### OMT-inflammation-itching target network construction and analysis

The 125 target genes in Figure 3A implicated in OMT, inflammation, and itch was built into a PPI network diagram using the String database, as illustrated in Figure 3B. The network had 125 nodes, 385 edges, a node degree of 6.21 on average, and a local clustering coefficient of 0.462 on average. The PPI results revealed a complex network of anti-inflammatory and antipruritic actions of OMT. The network diagram of the “OMT-inflammation-pruritus” target was constructed and shown by Cytoscape 3.7.1 software to describe the complicated network interaction of OMT anti-inflammatory and antipruritic more simply and intuitively, as shown in Figure 3C. Figure 3D showed the fraction of genes in the network, with HSP90AA1, SRC, ESR1, GRB2, and RXRA as the key genes. OMT interacts with several targets, as shown in the diagram in Figure 3E, reflecting the intricate network of multi-target interactions prevalent in traditional Chinese medicine.

### The GO and KEGG enrichment analysis of potential anti-inflammatory and antipruritic targets of OMT

**FIGURE 4:**
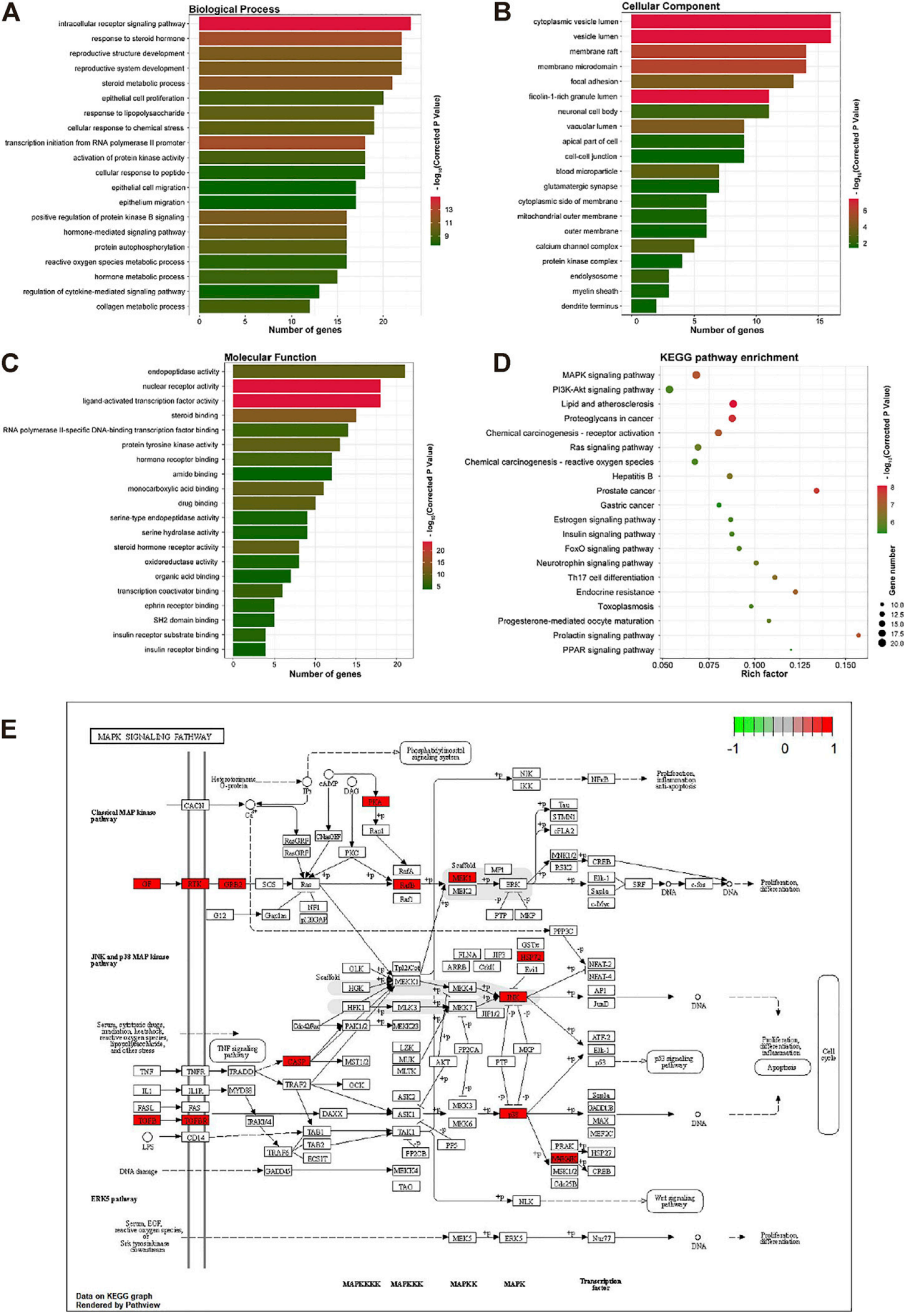
The GO and KEGG enrichment analysis of the common targets of OMT, inflammation, and itch. **(A–C)** GO enrichment analysis of the biological process, molecular function, and all components; *X*-axis for gene number and *p*-value for color. **(D)** KEGO enrichment analysis of the Common targets of OMT, inflammation, and itch. *Y*-axis for pathway, *X*-axis for gene number and *p*-value for color. **(E)** MAPK signaling pathway in the anti-inflammatory and antipruritic effect of OMT. Pathway analysis of the common targets of OMT, inflammation, and itch. The functional analysis of genes was enriched in the KEGG signaling pathway. The MAPK signaling pathways in the anti-inflammatory and antipruritic effect of OMT were highlighted in red.

To reveal the action of OMT on inflammation and itching, the biological process (BP), cellular component (CC), and molecular function (MF) were analyzed. The biological processes, components, and molecular activities of common targets are the primary focus of GO enrichment analysis. A total of 125 common targets were enriched in BP, CC, and MF, and the top 20 with the highest relevance (*p* < 0.05) were selected listed in Figures 4A–C. The results showed that the main BP terms were the reaction to steroid hormones, the response to peptides, and the positive regulation of the MAPK cascade the biological processes that were most abundant; The CC mainly were enriched in the cytoplasmic cavity, cyst cavity, secretory granule cavity. The main MF terms were the endopeptidase activity, steroid hormone receptor activity, nuclear receptor activity, transcription factor activity, and direct control of ligand sequence-specific DNA binding. In the KEGG pathway enrichment, MAPK signaling pathway, PI3K-AKT signaling pathway, proteoglycan in cancer, Ras signaling pathway, hepatitis B, and other pathways mostly occupied the forefront, indicating that OMT may treat itching by suppressing inflammation-related signaling pathways. Therefore, this study focused primarily on the MAPK signaling pathway in OMT’s anti-inflammatory and antipruritic properties. MAPK10, MAPK8, MAPKAPK2, HSPA8, MAPK14, MET, PGF, ERBB4, PRKACA, TGFR1, TGF2, FGFR1, KDR, BRAF, MAP2K1, CASP3, GRB2, TEK, KIT, INSR are among the targets shown in Figures 4D,E.

### WGCNA analysis of potential target genes related to atopic dermatitis

**FIGURE 5:**
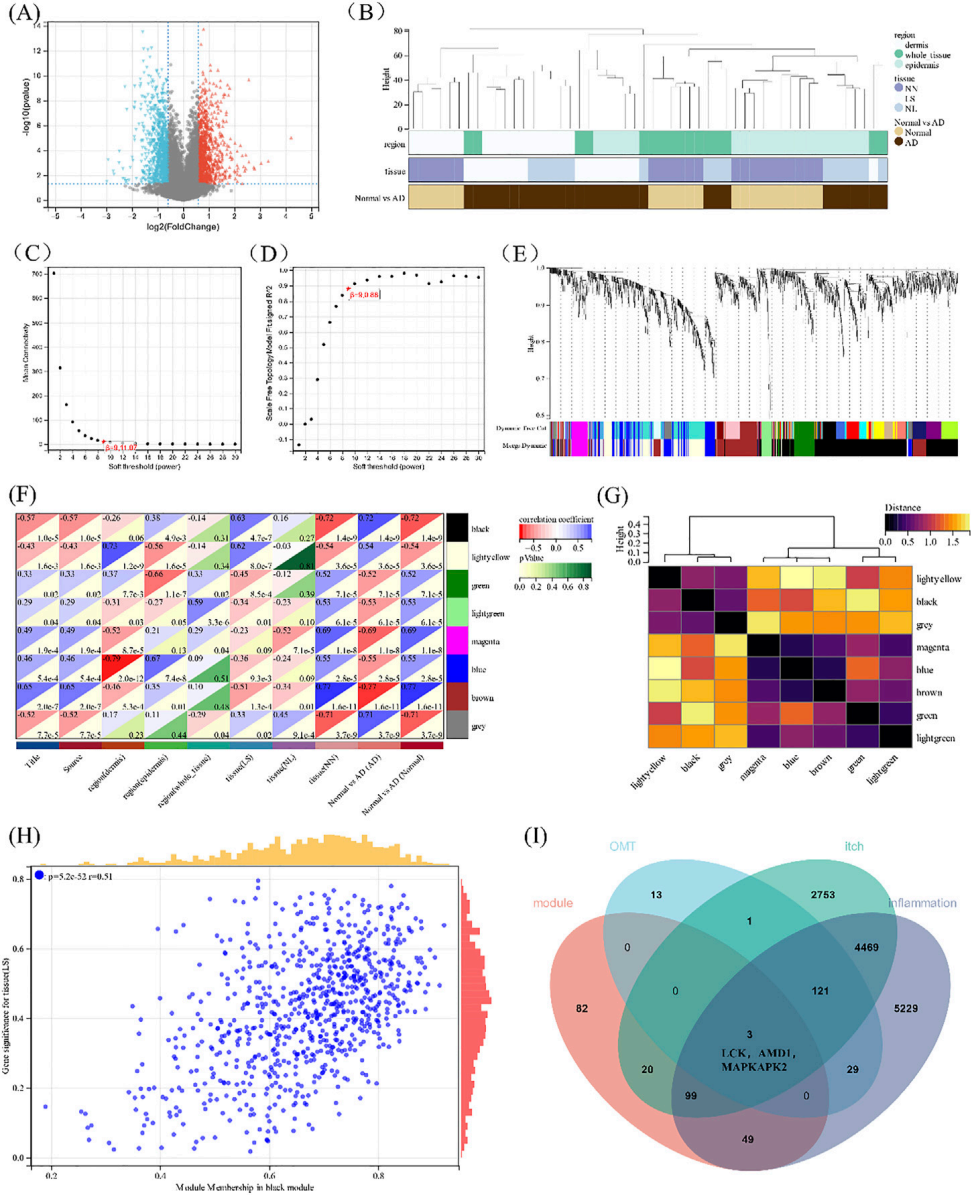
Construction of WGCNA. **(A)**The volcano plot of differentially expressed genes between atopic dermatitis and normal samples. **(B)** Clustering dendrogram of samples. **(C,D)** Analysis of the scale-free fitting indices for various soft-thresholding powers (β) and mean connectivity analysis of various soft-thresholding powers. **(E)** Clustering dendrogram of genes based on the measurement of dissimilarity (1-TOM). **(F)** The heat map of correlation between the module eigengenes and atopic dermatitis. **(G)** Heat map of the module eigengenes. **(H)** Scatter plot of module tissue (LS) in the black modulo. **(I)** Venn diagram of the black and yellow module eigengenes, the target of 0MT, itching, and inflammation.

To accurately reflect the characteristics of itching, the atopic dermatitis clinical dataset GSE120721 from the GEO was adopted to show the differential gene analysis, and 2127 differential genes were obtained utilizing shown in Figure 5A. All samples were in the clusters and within the cut-off threshold value (height < 80), and three clinical variables (disease status, tissue, and region) were adopted in the WGCNA as shown in Figure 5B. Furthermore, the soft threshold is set to 9 (Figure 5C) to make the created network more by the features of a scale-free network. To produce distinct gene modules, hierarchical clustering analysis was done using a weighted approach, and segmentation was performed using the specified soft threshold and the clustering findings of clinical indications (Figures 5D,E). Based on normalization with soft threshold = 9, the number of genes inside a module n 30, and module cut, eight modules were determined. Normalization with soft threshold = 9, the number of genes inside a module *n* = 30, and module cut height 0.25 resulted in the identification of eight modules. Figure 5F depicts the eight modules, as well as atopic illness, severity, and depth of skin lesions. In addition, the GS scatterplot of the disease progression in atopic dermatitis (*p* < 0.001) MM in the black module was plotted in Figure 5H. The *p* values of the black and yellow panels are less than 0.001, indicating that they are highly correlated. Therefore, these two modules were selected for subsequent analysis.

When the 254 genes in these two modules were compared to drug-disease relevant genes, it was discovered that LCK, AMD1, and MAPKAPK2 shared three genes in common (Figure 5I). KEGG is linked to the MAPK signaling pathway when combined with the above drug-disease targets. As a result, these three genes were chosen for further molecular docking and experimental research, for which were thought to be involved in the occurrence and progression of the disease, are related to the MAPK pathway upstream of MAPKAPK2 and may become potential targets of OMT in the treatment of pruritus.

### Molecular docking of OMT with related target proteins

**FIGURE 6:**
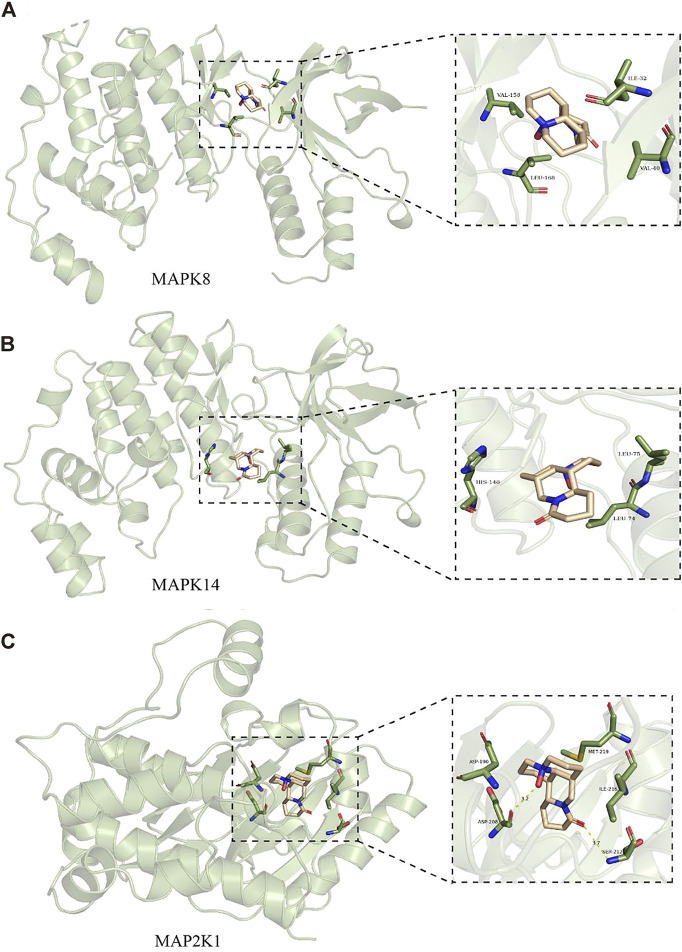
Molecular docking models of MAPK8, MAPK14, and MAP2K1. **(A)** are OBIT and MAPK8 docking mode and the interaction plane diagram. **(B)** are ONIT and NIAPKI4 docking model and the interaction plane diagram. **(C)** were OMT and MAP2K1 docking model and the interaction plane diagram.

Molecular docking was applied to validate the binding of OMT to 20 important target proteins which are shown in Table 3. The results showed that OMT is compatible with the structure of protein receptors, and it mostly binds to histidine, phenylalanine, leucine, tryptophan, and other amino acid residues in proteins *via* hydrogen bonds and π-π bonds interactions. AR, the docking protein with the highest score, attaches to amino acids like ASN705, LEU707/873, MET742/787, and PHE764 through hydrogen bonds and -bonds. MAPK8, MAPK14, and MAPK2K1, all of which are involved in the MAPK signaling pathway, had binding energies of −7.8, −7.9, and −8.4, respectively, indicating excellent binding (Figures 6A–C). The results indicated that OMT could be bound to the active site of the above targets.

**TABLE 3:** Affinities and amino acid sites of ligand-protein detected by molecular docking.

Protein	Ligand	Binding energy kcal/mol	Amino acid binding site
OMT	ABL1	−8.1	LEU248/370,TYR253,VAL256/299,LYS271
	ALB	−8.4	LEU398/529,LYS402,ALA406,VAL409,ASP549
	AR	−9.1	ASN705,LEU707/873,MET742/787,PHE764
	CASP3	−6	TRP206/214,ASN208,PHE250
	ESR1	−8.9	LEU346/384/387/391/525,ALA350,MET388,PHE404
	GRB2	−6.9	ILE85,PHE95,VAL110,TYR118,LEU120
	HSP90AA1	−7.8	MET98,LEU107,PHE138,TYR139
	HSP90AB1	−7.2	ASN51,ALA55,MET98,LEU107
	JAK2	−8.3	LEU855/983,VAL863,SER936
	LCK	−7.8	VAL259/301,ALA271/381,GLU288,MET292,THR316
	MAP2K1	−8.4	ASP190/208,SER212,ILE216,MET219
	MAPK14	−7.9	LEU74/75,HIS148
	MAPK8	−7.8	ILE32,VAL40/158,VAL158
	MDM2	−6	ILE57,MET58,TYR63,VAL71/89
	NR3C1	−8.2	GLY567,MET601/604/646,ALA605,LEU608,PHE623
	PRKACA	−7.8	LEU49/173,VAL57,ALA70,GLU127,PHE327
	PTPN11	−7.4	LYS358,GLU359,VAL360,HIS394,ASP395/431
	RXRA	−7.7	ILE268/310,ALA271/272,TRP305,LEU309/326,PHE313,CYS432
	SRC	−7.6	PHE278/307,GLU280/310,LYS295,LEU297,ILE336
	STAT1	−7.9	GLN275,GLU353,TYR1356,ASN1357

### The effect of OMT on the viability of RAW264.7 cells

**FIGURE 7:**
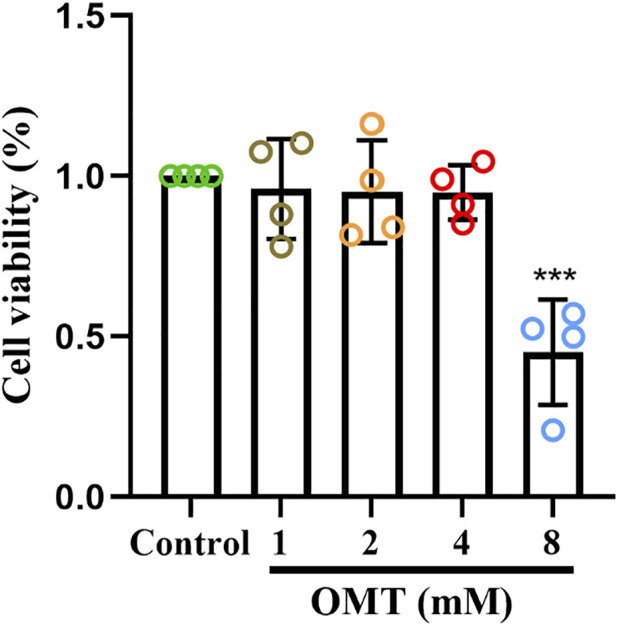
The effect of OMT on the viability of RAW264.7 cells. Cytotoxic effect of various concentrations of OMT (0, 1, 2, 4, and 8 mM) on RAW264.7 cells after 24 h examined by using the CCK8 assay. Values represent average + S.U. Significant differences among different groups am indicated as ***p < 0.001 vs. control group.

The CCK-8 kit was utilized to assess the effect of OMT administration alone on the viability of RAW264.7 cells to determine the safe administration range of OMT to RAW264.7 cells. The results revealed that OMT in concentrations below 4 mM did not affect RAW264.7 cell viability, but at 8 mM, viability was reduced by around 50% as shown in Figure 7. As a result, the safe dosing range of OMT to RAW264.7 cells is thought to be 0–4 mM. The low, medium and high administration concentrations of OMT were determined to be 25, 50, and 100 μM according to previous studies (Zhang et al., 2015).

### The effect of OMT on LPS-induced RAW264.7 cell in cell morphology

**FIGURE 8:**
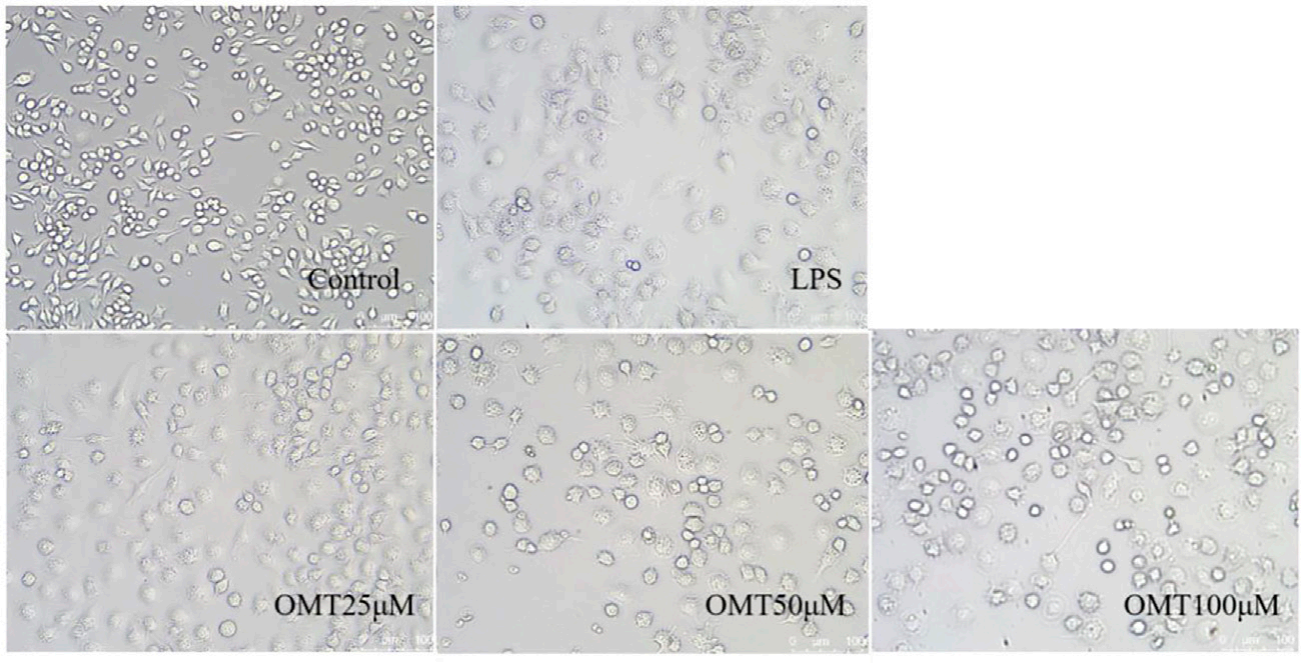
The effect of OMT on the cell morphology of LPS-induced RAW264.7 cell inflammation model. Each group was visualized by an ordinary light microscope (× 200 magnification).

**FIGURE 9:**
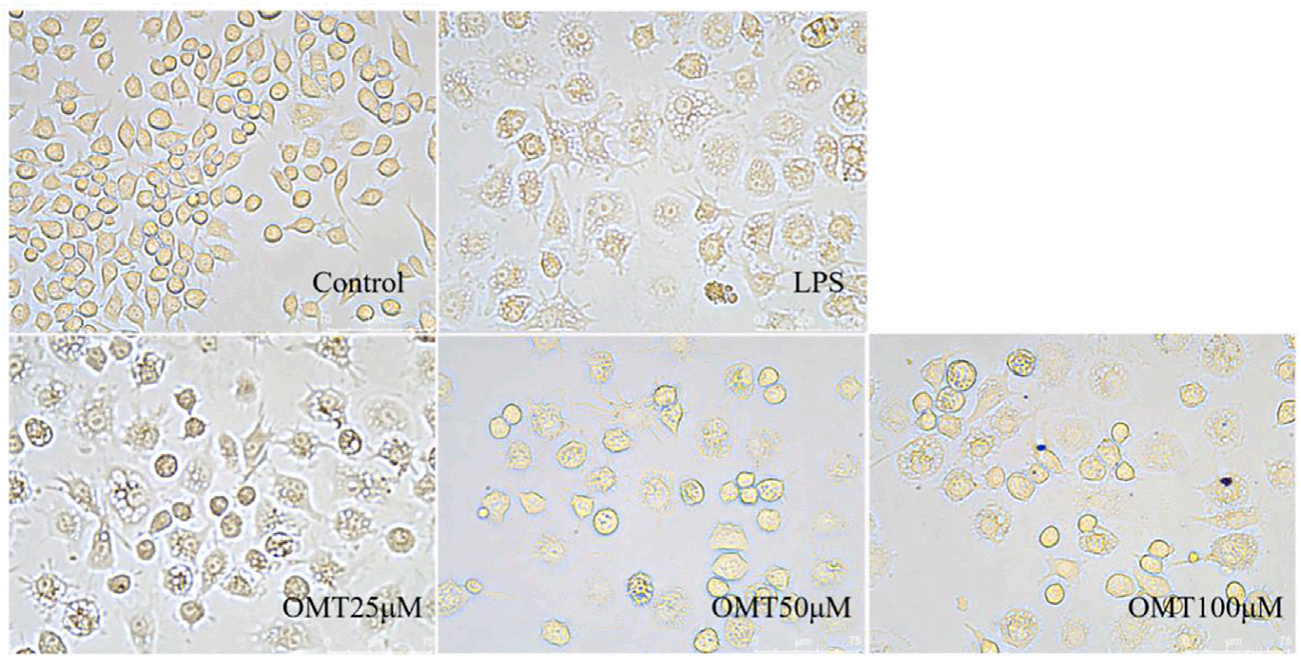
The effect of OMT on the cell momhology of LPS-induced RAW264.7 cell inflammation model. Each group was visualized by an ordinary light microscope (× 400 magnification).

Next, LPS-induced RAW264.7 cell inflammation model was adopted to investigated the effects of OMT on inflammation. The OMT medium was discarded, washed twice with PBS, and RAW264.7 cells were grown with 1 μg/ml LPS media for 24 h after being pre-protected with different doses of OMT for 1 h. As shown in Figure 8 and Figure 9, the RAW264.7 cells in the control group were smaller, spherical, and prismatic, however, after 24 h of LPS 1 μg/ml treatment, the morphology of the cells changed to a dendritic form, and the specific expression was the cell volume, after various amounts of OMT pre-protected, compared to the LPS group. The RAW264.7 cells in the control group were smaller, spherical, and prismatic, after 24 h of exposure to LPS 1 μg/ml, the morphology of the cells changed to a dendritic form, and the specific expression was the cell volume. The cell morphology was improved to varying degrees after different doses of OMT pre-protected the cells, and the LPS-induced RAW264.7 cell morphology was suppressed. The result showed that OMT exhibits inflammatory protective effect with a certain concentration gradient of 25, 50, 100 μM.

### The effect of OMT on LPS-induced RAW264.7 cell in apoptosis

**FIGURE 10:**
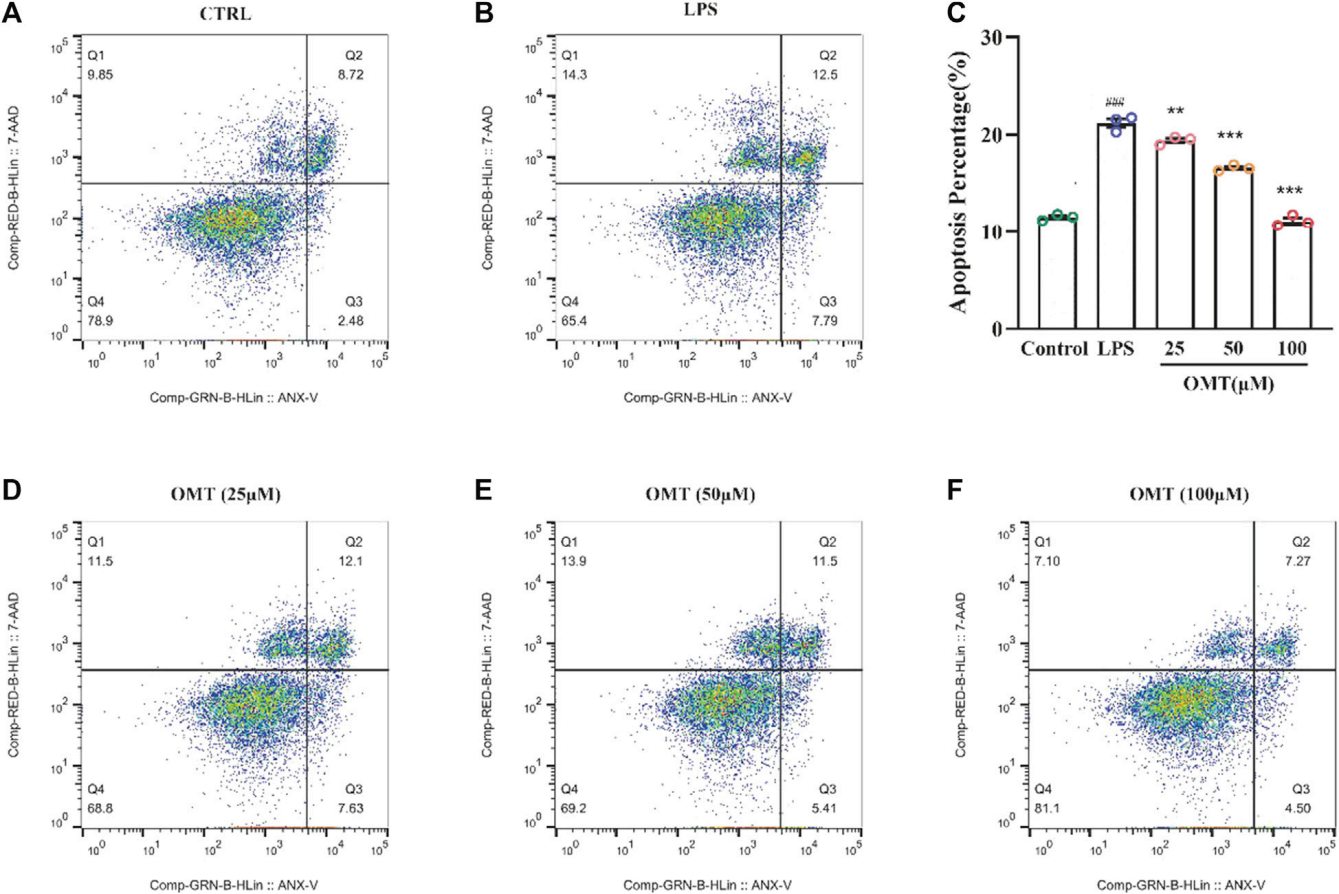
Effects of OMT on the apoptosis of LPS-induccd RAW264.7 cell inflammation model detected by flow cytometry and the histogram of apoptosis rate of cells. **(A)** Raw264.7 cells (control): live cells: 78.9%, necrotic: 9.85%, early apoptosis: 2.48%, late apoptosis: 8.72% **(B)** RAW264.7 cell+ LPS: live cells: 65.4%, necrotic: 14.3%, early apoptosis: 7.79%, late apoptosis: 12.5% **(C)** RAW264.7 cells + OMT (25 iM): live cells: 68.8%, necrotic: 11.5%, early apoptosis: 7.63%, late apoptosis: 12.1%. **(D)** RAW264.7 cells+ OMT (50 iM): live cells: 69.2%, necrotic: 13.9%, early apoptosis: 5.41%, late apoptosis: 4.50% **(E)** RAW264.7 cells + OMT (100 iM): live cells: 81.1%, necrotic: 7.10%, early apoptosis: 4.50%, late apoptosis: 7.27%. Values represent average ± S.D. Significant differences among different groups are indicated as ^###^ *p* <0.001 vs. control; *p < 0.05, **p <0.01, ***p <0.001vs. LPS group.

To investigate the protective effect of OMT on LPS-induced RAW264.7 cells, flow cytometry was applied for evaluation. Figure 10 showed that the apoptosis in an LPS-induced RAW264.7 cell inflammation model, the control group had an apoptosis rate of 11.2 percent, however, the LPS group had an apoptosis rate of 20.29 percent, which was considerably greater than the control group, which is significantly different (*p* < 0.05). RAW264.7 cells’ apoptosis rate was decreased to varying degrees following pre-protection with different concentrations of 25, 50, and 100 μM OMT as compared to the LPS group. The proportions were 19.73 percent, 16.91 percent, and 11.77 percent, respectively, indicating that there was a dosage dependency. These results showed that OMT exerted a protective effect on LPS-induced inflammation.

### The effect of OMT on the expression of MAPK signaling pathway in LPS-induced RAW264.7 cell

**FIGURE 11:**
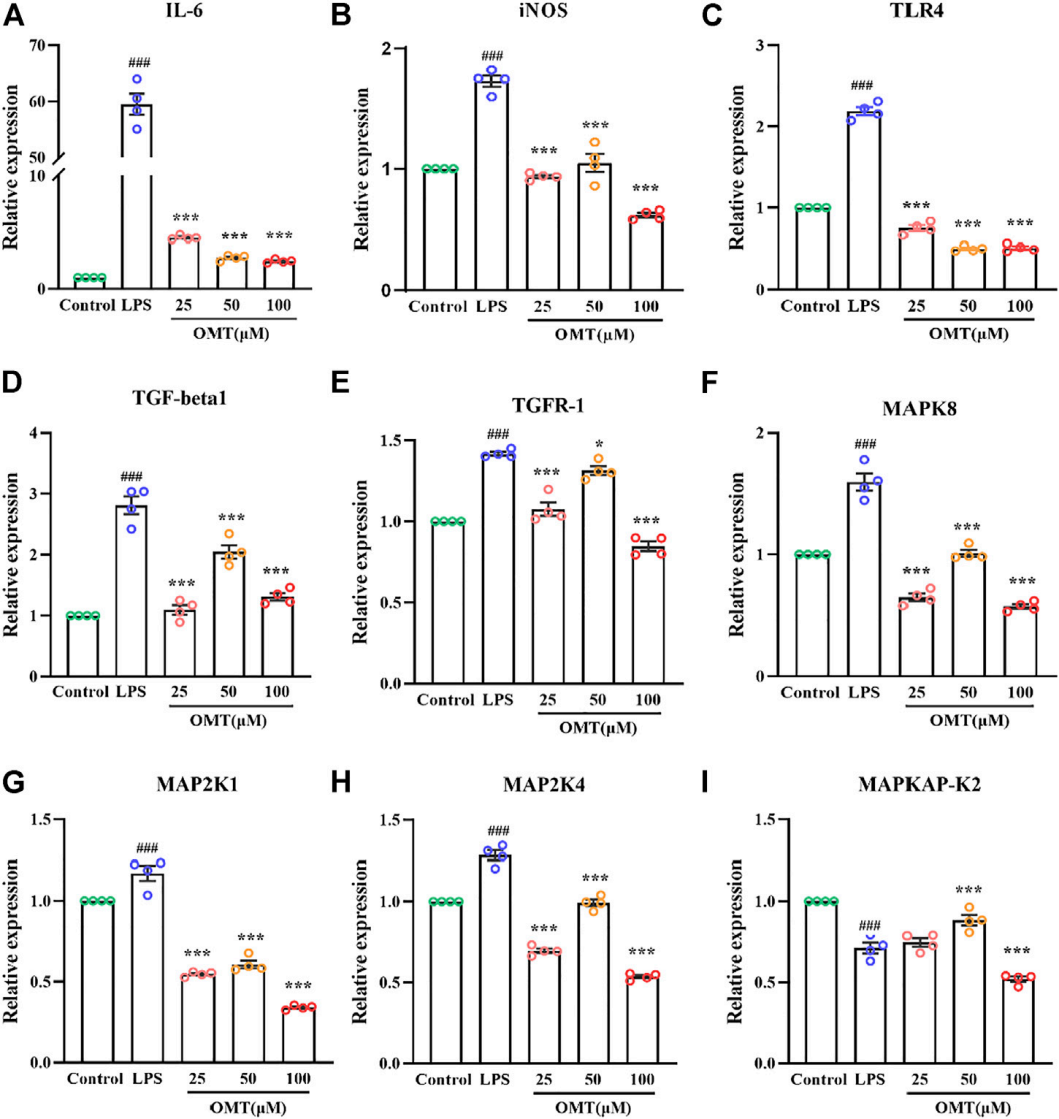
Effects of OMT on the mRNA expression of LPS-induccd RAW264.7 cell inflammation model. **(A–E)** The mRNA expression of inflammatory factors (IIL-6, iNOS), and receptors (TLR4, TOF-R-1, TGF-betal). **(F–I)** The mRNA expression of MARK signaling pathway targets (MAPK8, MAP2KI, MAP2K4, MAPKAP-K2). Values represent average ± S.D. Significant differences among different groups are indicated as ^###^ *p* < 0.001 vs. control; *p <0.05, **p < 0.01vs. ***p < 0.001 vs. LPS group.

RT-qPCR was used to identify the amounts of inflammatory components to validate whether the inflammation model was effective. As shown in Figure 11, RT-qPCR analysis of expression indicated that when LPS was cultured at 1 μg/ml for 24 h, the relative expressions of IL-6 and iNOS mRNA were dramatically raised, showing that the inflammatory model had been successfully generated. The pre-protective impact of different doses of OMT considerably prevented the rise in relative expression levels of IL-6 and iNOS mRNA induced by LPS as compared to the LPS group, indicating that OMT has a considerable anti-inflammatory effect. The relative expression of TLR4 mRNA was observed for LPS activates the TLR4 receptor. When cells were cultured with LPS 1 μg/ml for 24 h, the relative expression of TLR4 mRNA increased. Different concentrations of OMT were used in comparison to the LPS group. The relative expression of TLR4 mRNA produced by LPS was considerably decreased after the pre-protective impact of OMT, suggesting that OMT may suppress the action of LPS on TLR4 receptors. Furthermore, the relative mRNA expression levels of MAPK signaling pathway-related genes were found to be suppressed. Except for the MAPKAPK-2 gene, the other administration groups were able to considerably reduce the abnormally high production of gene mRNA produced by LPS, including TGF-beta1, TGFR-1, MAPK8, MAP2K1, and MAP2K4, suggesting that OMT may be involved. Through the MAPK signaling pathway, it has anti-inflammatory action.

### Effects of OMT on the mRNA, protein, and phosphorylation levels of p38 gene in LPS-induced RAW264.7 cell

**FIGURE 12:**
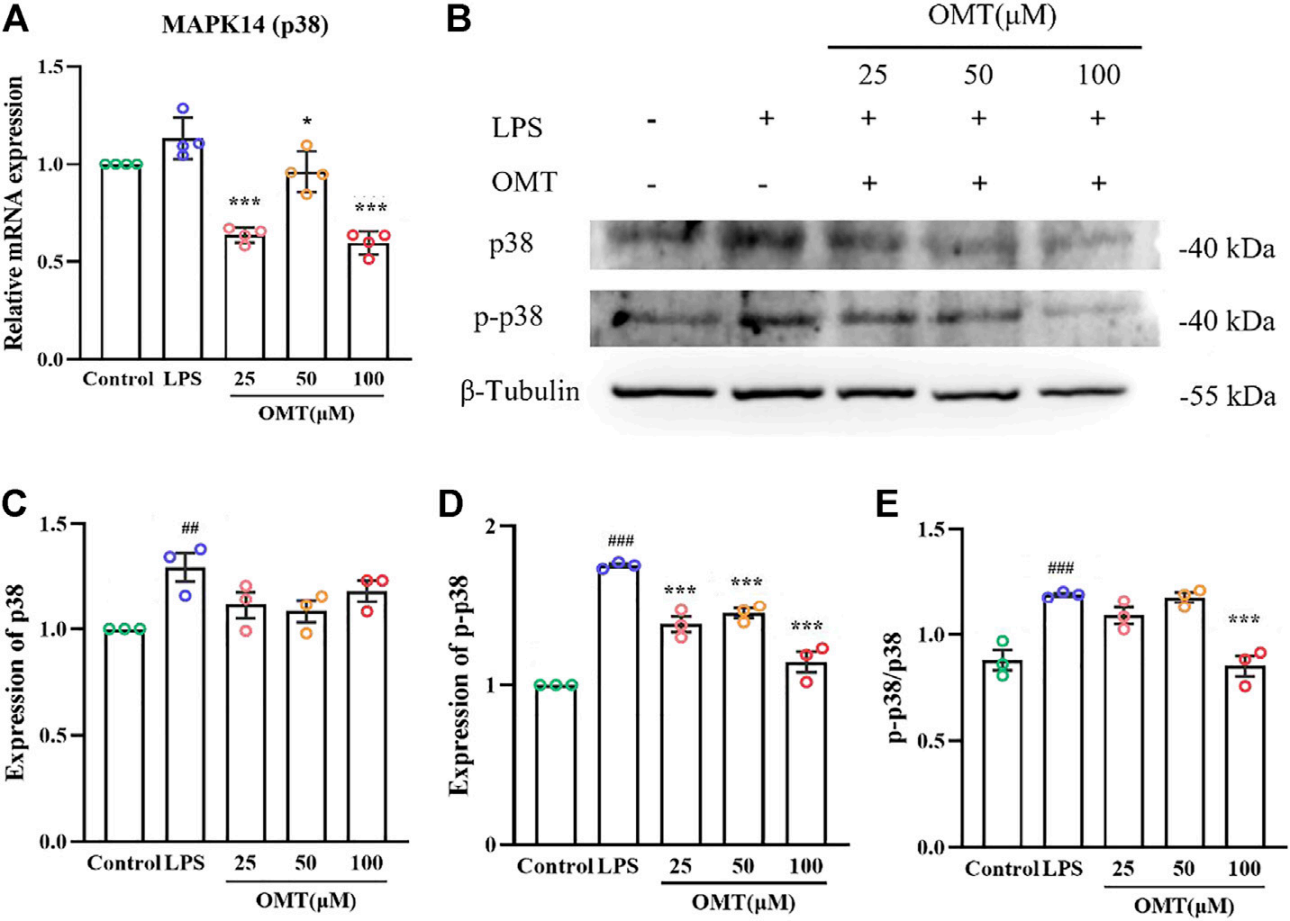
Effects of OM on the mRNA expression and protein phosphorylation expression of LPS-induced RAW264.7 cell inflammation model. **(A)**The mRNA expression of p38 mRNA, **(B–E)** protein level and phosphorylation level of p38. Values represent average ± S.D. Signilicani differences among different groups are indicated as ^##^ *p* < 0.01, ^###^ *p* < 0.001 vs. control; *p <0.05, **p < 0.01, ***p < 0.001 vs. LPS group.

To further verify the analysis results, we conducted the following experiments. MAPK14 (p38) is a major MAPK signaling pathway target gene and in the LPS-induced RAW264.7 cell inflammation model, the effect of OMT on the mRNA, protein, and phosphorylation levels of the p38 gene was investigated. As shown in Figure 12, the RAW264.7 cells exerted no significant difference in the relative expression of p38 mRNA when compared to LPS 1 μg/ml for 24 h, while the 25, 50, and 100 μM OMT groups significantly decreased the relative expression of p38. Subsequently, the protein levels of p38 and p-p38 were determined. The results indicated that after 24 h of LPS 1 μg/ml treatment, the protein levels of p38 and p-p38 in RAW264.7 cells were facilitated compared to the blank control group. The expression of p38 protein of the group did not differ substantially from that of the blank control and the LPS group, however, the p-p38 protein level was considerably lower than that of the LPS group and the ratio of p38/p-p38 represented the amount of phosphorylation of p38. The phosphorylation level of p38 in RAW264.7 cells was significantly increased after 24 h of exposure to 1 μg/ml LPS, whereas 100 μM OMT pre-protection significantly reduced the level of p38. These results suggested that OMT may have an anti-inflammatory effect by inhibiting p38 phosphorylation.

## Discussion

### Resource identification initiative

Many skin disorders such as atopic dermatitis, psoriasis, dry skin diseases, liver diseases, kidney diseases, and metabolic diseases are characterized by significant itching sensations. These disease types are mostly non-histamine-dependent pruritus (Cevikbas and Lerner, 2020). Pruritus can be classified as histamine-dependent or histamine-independent based on the involvement of histamine in the development of the itch. It can help with the symptoms of histamine-dependent itching, but it won't help with refractory itching (Paus et al., 2006; Johanek et al., 2007).

Macrophages of innate immune cells play an important role in the development of chronic pruritus. Which contribute to histamine-independent pruritus through chronic inflammation and are one of the main reasons for maintaining chronic inflammation and neural sensitization. Although corticosteroids can be used for therapy, they are easy to abuse and long-term usage can result in major side effects such as skin thickening, substance metabolism issues, rebound after medication withdrawal, and even skin itching (Elmariah and Lerner, 2011).

OMT is one of the main active ingredients of *Sophora flavescens* and is commonly used for analgesia, anti-inflammatory, and anti-itching in the acute phase of the disease. According to previous research (Xu et al., 2018), OMT exerts antipruritic and anti-inflammatory actions in ACD mice by modulating inflammatory mediators and restoring Th1/Th2 and Th17/Treg immunological balance. However, the impact on innate immune cells and the mechanism of action in neutrophils and macrophages are unclear, prompting more research.

Therefore, in our study, we analyzed the common targets of OMT by utilizing network pharmacology. The results of PPI analysis and GO analysis showed that OMT, regulats inflammatory response and related signal transduction, including MAPK signaling pathway, the PI3K-AKT signaling pathway, and others heavily represented in the KEGG pathway. To study the OMT targets with a more holistic approach, we used the GEO database to screen differentially expressed genes in Atopic dermatitis cases and conduct a WGCNA analysis. Atopic dermatitis is a chronic, inflammatory skin disease characterized by pruritus and recurrent eczematous lesions (Otsuka et al., 2017). To treat the illness, a variety of therapeutic options are available, including topical treatments, systemic medications, and biologics. Despite this, due to the chronic nature of many patients’ illnesses and their repeated episodes, care can be difficult (Folster-Holst et al., 2022).

The results showed that 2127 differential genes were separated into 8 modules, and three gene groups closely connected to the occurrence and development of atopic dermatitis were mined in this study using the WGCNA approach coupled with network pharmacology to design a scale-free network. Furthermore, we further verified that the selected MAPK signaling pathway such as MAPK8, MAPK14, MAPK2K1, and MAPKAP-K2 was associated with OMT binding through molecular docking technology combined with network pharmacology analysis and WGCNA analysis. LCK, AMD1, and MAPKAPK2 might be possible oxymatrine targets in the treatment of pruritus. The results of molecular docking demonstrate that OMT is compatible with the structure of protein receptors, including MAPK8 and other MAPK signaling pathway targets. MAPK14 and MAP2K1 had binding energies of −7.8, −7.9, and −8.4, respectively, indicating a good binding impact. These findings suggest that OMT is a multi-signaling system that inhibits inflammation, oxidative stress, and other processes while also acting as an antipruritic.

This suggested that OMT affected the inflammatory response of macrophages *via* regulating the MAPK signaling pathway. In the development of chronic pruritus, various factors lead to the activation of the MAPK pathway of inflammatory cells to cause pruritus and neural sensitization. How does activation of the TLR4/MAPK pathway lead to the development of chronic pruritus and neural sensitization? The MAPKs are a family of serine/threonine protein kinases that can be triggered by a variety of external stimuli (such as cytokines, neurotransmitters, etc.). MAPK kinase kinases, MAPKs kinases, and MAPKs are the fundamental components of the MAPK signaling pathway, which is largely conserved from yeast to humans. These three kinases can all be active at the same time to control cell development, differentiation, stress response, inflammatory response, and other important biological processes. Extracellular signal-regulated protein kinase (ERK), c-Jun N-terminal kinase (JNK), p38 mitogen-activated protein kinase (p38 MAPK), and extracellular signal Regulatory Kinase 5 (ERK5) are the four main branches of the MAPK pathway (Sun et al., 2015). JNK and p38 MAPK signaling pathways, for example, have comparable activities and are involved in stress responses including inflammation and apoptosis (Yeung et al., 2018; Hammouda et al., 2020). Moreover, LPS constantly activates a crucial axis formed by reciprocal crosstalk between the p38 (MAPK) pathway and signal transducer and activator of transcription (STAT) 3-mediated signal transduction. The production and proliferation of inflammatory macrophages dependent on homeostatic activation of this axis (Bode et al., 2012).

In our study, after different doses of OMT pre-protected the LPS-induced inflammatory model of mouse macrophages (RAW264.7 cells), the cell morphology improved to various degrees, and the morphological alterations of LPS-induced RAW264.7 cells were blocked, according to flow cytometry data. The fraction of apoptotic RAW264.7 cells dropped to varying degrees, and the LD content induced by LPS was greatly reduced. These results demonstrated that OMT exerted a protective effect on LPS-induced inflammatory apoptosis. In addition, OMT can inhibit the release of inflammatory factors and the expression of inflammatory factor receptors in LPS-induced RAW264.7 cells. The mitogen-activated protein kinase (MAPK) family includes p38 MAPK. P38 MAPK residues Thr180 and Tyr182 are phosphorylated by p38-specific MKK3 and MKK6 in the p38 MAPK signaling cascade. When p38 MAPK is phosphorylated, it undergoes a conformational shift, allowing the substrate to access the catalytic residue. The functions of the p38 MAPK signaling pathway have been mediated by more than 60 downstream substrates so far (Trempolec et al., 2013). P38 MAPK is divided into four isoforms, each of which is encoded by a separate gene (Jiang et al., 1997). In monocytes and macrophages, pharmacological investigations have demonstrated that several drugs that selectively inhibit isoforms have anti-inflammatory effects. Since p38 is highly expressed in these cells, it is thought that p38 is primarily involved in the inflammatory response. P38 MAPK, which can be activated by upstream pro-inflammatory cytokine receptors and produce downstream signaling molecules to regulate macrophages, has multiple roles in the inflammatory response. The inflammatory response is influenced by several biological mechanisms involving cells and other immune cells. A recent study has shown the potential of the p38 MAPK-MK2 signaling axis as a target for therapeutic intervention in many neurological diseases (Beamer and Correa, 2021).

RT-qPCR was also utilized to assess inflammatory factor levels, and the pre-protective impact of different doses of OMT considerably prevented the rise in the mRNA expression of inflammatory factor IL-6, INOS, and the inflammation relative receptor TLR4, TGF-beta1, TGFR-1 induced by LPS. Moreover, OMT also suppresses downstream of the MAPK signaling pathway-associated genes’ relative mRNA expression levels. The findings revealed that, in addition to the MAPKAPK-2 gene, OMT significantly reduced abnormally high mRNA expression of genes produced by LPS, such as TGF-beta1, TGFR-1, and MAPK8, MAP2K1, and MAP2K4.

Finally, the activity of phosphorylation sites in the MAPK pathway was examined and the facilitation of phosphorylation of P-38 protein induced by LPS was inhibited, indicating that OMT takes functional regulation of macrophages *via* the MAPK signaling pathway.

## Conclusion

OMT interacts with a multitude of targets and possesses anti-inflammatory and antipruritic properties that are multi-channel, multi-link, multi-target, and overall synergistic. In addition, the MAPK signaling network was mostly enriched when WGNCA, molecular docking, and KEGG pathway enrichment analysis were combined with *in vitro* experimental results, suggesting that OMT may exert anti-inflammatory and antipruritic effects by affecting the MAPK signaling pathway, providing a reference for future mechanism research.

## Data Availability

The datasets presented in this study can be found in online repositories. The names of the repository/repositories and accession number(s) can be found in the article/Supplementary Material.
